# Design, synthesis, and anti-*Toxoplasma gondii* evaluation of β-carboline derivatives

**DOI:** 10.1186/s13071-025-07139-6

**Published:** 2025-12-29

**Authors:** Zhendi Liu, Yongmei Li, Yetian Li, Xiaoyu Han, Hongda Qiu, Chang Qin, Yuchao Zhu, Weida Liang, Jiao Mo, Zixun Yan, Weixin Gao, Jiyu Zhang, Jishan Zheng, Hongze Liang, Jili Zhang

**Affiliations:** 1 School of Basic Medical Sciences, Health Science Centre, Ningbo University, Ningbo, 315211 China; 2 Key Laboratory of Advanced Mass Spectrometry and Molecular Analysis of Zhejiang Province, School of Materials Science and Chemical Engineering, Ningbo University, Ningbo, 315211 China; 3 Lanzhou Institute of Husbandry and Pharmaceutical Sciences, Chinese Academy of Agricultural Sciences, Lanzhou, 730046 China; 4 Department of Radiology, First Affiliated Hospital of Ningbo University, Ningbo, 315010 China; 5 Ningbo Women and Children’s Hospital, Ningbo, 315012 Zhejiang China

**Keywords:** *Toxoplasma gondii*, β-carboline derivatives, *In vitro*, *In vivo*

## Abstract

**Background:**

The current therapeutic options for toxoplasmosis are limited by side effects. The development of molecules against *T. gondii* is urgently needed. A series of β-carboline derivatives were synthesized and examined as potential agents against toxoplasmosis.

**Methods:**

A series of β-carboline derivatives were synthesized. To assess their potential as anti-*T. gondii* agents, cytotoxicity towards Vero cells was determined using the CCK-8 assay. Plaque and qPCR assays were carried out to screen for anti-*T. gondii* activities, providing insights into their inhibitory effects on the parasite. *In vitro* assays on *T. gondii* RH and PRU strains were conducted to evaluate proliferation, invasion, and cyst formation. Transmission electron microscopy was employed to analyze ultrastructural changes and apoptosis in *T. gondii*, revealing the impact of the derivatives at the cellular level. Finally, the *in vivo* efficacy of the derivatives was tested in a mouse model, which offered valuable information on their potential therapeutic effects in a living organism.

**Results:**

β-carboline derivatives exhibited potent inhibitory effects on the growth and replication of both PRU and RH strains, while demonstrating low cytotoxicity to mammalian cells. It is worth noting that NBZ023 and NBZ035 exhibited optimal potency against proliferation (IC_50_ = 2.85 and 1.6 μM) or invasion (IC_50_ = 4.72 and 1.13 μM) of *T. gondii*. Importantly, NBZ023 and NBZ035 had an effect on preventing PRU cysts formation, and they markedly reduced parasite burden in the brain, spleen, and liver in mouse infection model.

**Conclusions:**

Lead compounds NBZ023 and NBZ035 exhibited an excellent overall efficacy against the *T. gondii* RH and PRU strains, and were highly effective at preventing toxoplasmosis during murine infection, which are expected to be developed as new anti-toxoplasmosis drugs.

**Graphical Abstract:**

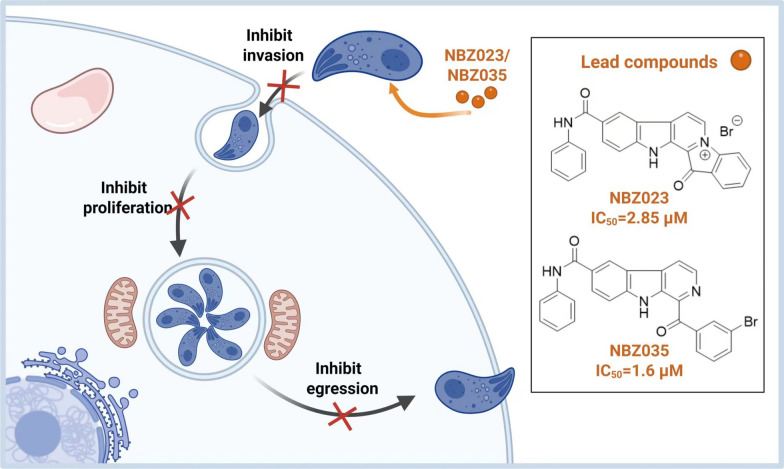

**Supplementary Information:**

The online version contains supplementary material available at 10.1186/s13071-025-07139-6.

## Background

*Toxoplasma gondii* (*T. gondii*) is an obligate intracellular protozoan parasite capable of infecting humans and nearly all warm-blooded animals, leading to the zoonotic disease toxoplasmosis [1]. Nearly one-third of the global population is infected by *T. gondii*, and seropositivity in some areas is approximately 70% [2]. Although primary infections are asymptomatic in immunocompetent individuals, *T. gondii* can persistently form tissue cysts that are mainly located in the brain, eyes, and striated muscle at the bradyzoite stage [3], and chronic toxoplasmosis is associated with attention-deficit hyperactivity disorder, autism, suicidal ideation, and suicide attempts [4–6]. Additionally, *T. gondii* infection often leads to severe symptoms or even death in immunocompromised patients [7]. When infection occurs in the early stages of pregnancy, *T. gondii* can be transmitted to the fetus through the placenta or birth canal, resulting in fetal malformation, miscarriage and even stillbirth [8–10]. Notably, treatments for acute toxoplasmosis are currently based on the combination of pyrimethamine and sulfadiazine, but this traditional therapeutic regimen is not always suitable for prolonged treatment because of its undesirable side effects, such as teratogenesis, cytopenia and allergic skin reactions [11, 12]. Currently, the folic acid synthesis inhibitors trimethoprim and sulfamethoxazole and the protein synthesis inhibitors spiramycin and azithromycin are also used for clinical toxoplasmosis, but they are ineffective in inhibiting cyst formation [12]. Atovaquone, which targets mitochondrial cytochrome bc1, inhibits the formation of bradyzoite cysts by *T. gondii*, but its clinical use is limited by its low solubility and bioavailability [13]. Owing to the complexity of the lifecycle of *T. gondii*, vaccines are unable to prevent and treat toxoplasmosis in clinical practice [14]. As safe and effective anti-*T. gondii* drugs are lacking, designing and synthesizing novel anti-*T. gondii* drugs, with promising targets, are expected to overcome the bottleneck of toxoplasmosis treatment on humans and animals.

β-Carboline alkaloids are widely found in marine organisms, land plants, cyanobacteria, mushrooms and the body fluids and tissues of mammals. Currently, various classes of natural and semisynthetic analogues of β-carbolines, such as those with hypnotic, sedative, anticonvulsant, anxiolytic, antiviral, antitumour, antimicrobial and antiparasitic effects, have received extensive attention because of their diverse biological and pharmacological effects [15]. The skeleton structure of β-carboline alkaloids has the advantages of simplicity, ease of synthesis and modification, good oral bioavailability, and a long half-life, making β-carbolines lead compounds with broad potential in the research and development of new drugs [16]. Alomar et al. reported that the β-carboline alkaloid derivatives norharmane, harmane, and harmine exerted anti-*T. gondii* effects by partially inhibiting *T. gondii* invasion and proliferation [17]. Further study suggested that harmine and its derivatives could cause cell cycle arrest in *T. gondii* and might act on the DNA repair process [18]. Accordingly, β-carboline alkaloids may be potential anti-*T. gondii* compounds [19]. Unfortunately, despite numerous biological activities of β-carboline derivatives, their anti-*T. gondii* activity needs to be improved, and their severe cytotoxicity caused by planar skeleton remain not effectively resolved.

To improve the safety and efficacy of β-carboline alkaloids, we designed a series of β-carboline derivatives substituted at the C6 moiety with hydrogen, methyl, carboxyl, benzoyl, or C1 moiety with halogen, methoxy. This study aims to further enhance the activity of β -carboline derivatives against *T. gondii* RH strain and PRU strain, which is anticipated to be beneficial for the future development of new β-carboline derivatives based anti-*T. gondii* therapeutics.

## Methods

### General

All the ^1^H NMR (400, 500, or 600 MHz) and ^13^C NMR (100, 126 or 151 MHz) spectra were acquired using a Bruker Avance II 400 MHz spectrometer (Bruker Corporation (BRKR), USA), a Bruker Ascend 500 MHz spectrometer or a Bruker Ascend 600 MHz spectrometer in DMSO-*d*_*6*_. High-resolution mass spectra (HRMS) were recorded on a Thermo Fisher LTQ Orbitrap XL spectrometer, an Agilent 6520 QTOF mass spectrometer (Agilent Corp., USA) or a Bruker microTOF-Q II system via electrospray ionization (ESI) in positive mode. All the compounds were exhibited > 98% pure as determined by HPLC analysis. The purity analysis method and results for compounds NBZ001‒026 and NBZ028‒036 were consistent with prior descriptions [19–23].

### General procedure for the synthesis of derivatives

Compounds NBZ001‒026, NBZ028‒036 were synthesized via previously described procedures [19–23], as shown in Scheme S1 and S2 [19–23]. The synthesis of the new compounds NBZ027 and NBZ037 is depicted in Scheme S3. The spectroscopic characterization of the new compounds NBZ027 and NBZ037 is shown in Figure S1.

(2-Chlorobenzoyl)-N-(2-methoxyphenyl)-9H-pyrido[3,4-b]indole-6-carboxamide (NBZ027): pale yellow solid, yield: 71%, m.p.: 228–230 °C. ^1^H NMR (600 MHz, DMSO) δ 12.50 (s, 1H), 9.50 (s, 1H), 9.05 (s, 1H), 8.58 (d, J = 4.9 Hz, 1H), 8.52 (d, J = 4.8 Hz, 1H), 8.25 (d, J = 8.6 Hz, 1H), 7.92 (d, J = 8.6 Hz, 1H), 7.85 (d, J = 7.7 Hz, 1H), 7.64 (d, J = 8.9 Hz, 1H), 7.58 (d, J = 8.7 Hz, 2H), 7.51 (t, J = 6.4 Hz, 1H), 7.22–7.17 (m, 1H), 7.12 (d, J = 8.1 Hz, 1H), 7.00 (t, J = 7.6 Hz, 1H), 3.87 (s, 3H). ^13^C NMR (151 MHz, DMSO) δ 196.05, 165.03, 151.28, 143.69, 138.99, 138.46, 135.69, 135.67, 131.35, 131.10, 130.03, 129.49, 129.25, 128.49, 126.99, 126.87, 126.70, 125.42, 124.08, 122.10, 120.14, 119.99, 119.67, 112.76, 111.26, 55.63. HRMS (positive mode): m/z calculated for C_26_H_18_ClN_3_O_3_: 456.1109 [M + H]^+^; found: 456.1101.

13-oxo-9-(p-tolylcarbamoyl)-12,13-dihydropyrido[1,2-a:3,4-b’]diindol-5-ium chloride (NBZ037): red solid, yield: 81%, m.p. (decomposition) > 300 °C. ^1^H NMR (400 MHz, DMSO-*d*_6_) δ 13.68 (s, 1H), 10.33 (s, 1H), 9.63 (s, 1H), 9.14 (s, 1H), 8.43 (s, 1H), 8.32 (s, 1H), 8.00 (d, J = 15.9 Hz, 2H), 7.80 (s, 1H), 7.66 (s, 3H), 7.14 (s, 2H), 2.25 (s, 3H). ^13^C NMR (151 MHz, DMSO) δ 181.50, 164.02, 147.75, 146.33, 140.14, 136.72, 135.74, 132.63, 131.31, 131.13, 128.63, 126.71, 125.23, 123.77, 123.26, 122.22, 120.71, 120.26, 119.88, 118.55, 115.18, 112.94, 20.01. HRMS (positive mode): m/z calculated for C_26_H_18_ClN_3_O_2_: 404.1394 [M]^+^; found: 404.1384.

## Materials

African green monkey kidney (Vero) cells were cultured in Dulbecco’s modified Eagle’s medium (DMEM) supplemented with 100 U/mL penicillin, 100 μg/mL streptomycin, 1% nonessential amino acids (NEAAs), 1% GlutaMAX and 10% fetal bovine serum (FBS) at 37 °C in an atmosphere of 5% CO_2_.

The *T. gondii* RH and PRU strains that were used in our study were generously donated by the Lanzhou Institute of Husbandry and Pharmaceutical Sciences, China. *T. gondii* RH tachyzoites were cultured in monolayer Vero cells, as described in a previous study [24]. *T. gondii* RH strain parasites constitutively expressing nuclear green fluorescent protein (RH-GFP) were maintained in Vero cells.

Compounds NBZ001‒037 were dissolved in dimethyl sulfoxide (DMSO; Sigma, USA) to a concentration of 12 mM and subsequently diluted with DMEM containing 1% FBS to various concentrations. Pyrimethamine was used as the positive control in *in vitro* experiments, and was dissolved in DMEM supplemented with 1% FBS to a final concentration of 0.4 μM. NBZ023 and NBZ035 were dissolved in isotonic saline containing 5% ethanol and 5% Cremophor EL to a concentration of 1 mg/mL for *in vivo* experiments. Sulfadiazine (10 mg/mL, Sigma, USA), pyrimethamine (5 mg/mL, Sigma, USA), and folinic acid (1.5 mg/mL, Sigma, USA) were suspended in isotonic saline containing 1% CMC-Na and used as positive drugs in *in vivo* experiments.

### Cell viability assay

Vero cells (1 × 10^4^ cells/well) were seeded in 96-well plates and treated with compounds NBZ001‒037 at concentrations ranging from 384 pM to 30 µM. DMEM without a drug was used as a control. After 24 h of treatment, Cell Counting Kit-8 (CCK-8, CA1210, Solarbio) was used to determine cell viability at 450 nm via a Multiskan GO instrument [24, 25].

### Screening of the compounds for Anti-*T. gondii* activity *in vitro*

The anti-*T. gondii* activity of compounds NBZ001‒037 was examined via RT-PCR. Confluent Vero cells in 6-well plates were infected with 2 × 10^5^ *T. gondii* RH tachyzoites per well, incubated for 6 h, and then washed twice with PBS to remove extracellular tachyzoites. The cell monolayers were incubated with DMEM (1% FBS) containing various concentrations of NBZ001‒037 for 24 h. Total genomic DNA from each sample was isolated with the DNAiso reagent (Takara), and the levels of the 529-bp repeat element of *T. gondii* were measured by qPCR. A plaque assay was used to evaluate the activity of the compounds. The detailed experimental procedures were described in previous studies [25, 26].

### *In vitro* anti-proliferation and anti-invasion assay with *T. gondii* RH strain tachyzoites

The antiproliferative effects of NBZ023 and NBZ035 on *T. gondii* were determined via qPCR, which was performed by using a QuantStudio 3 Flex real-time PCR system (Life Technologies). Cells were infected with tachyzoites (2 × 10^5^ per well) for 6 h, then treated with NBZ023, NBZ035 (0.24‒30 μM) or pyrimethamine (0.4 μM) for 24 h, and washed twice with PBS. Infected or uninfected cells that were cultured with DMEM (1% FBS) were used as the parasite or the host cell control group, respectively. Total genomic DNA from the cell samples was isolated with the DNAiso reagent (Takara), and the levels of the 529-bp repeat sequence of *T. gondii* were measured via qPCR as before.

Vero cells were seeded onto 6-well plates and cultured with DMEM supplemented with 10% FBS to obtain cell monolayers. The medium was replaced with fresh DMEM (1% FBS) containing NBZ023 or NBZ035 (0.24–30 μM), pyrimethamine (0.4 μM) or no drugs (as a control), and tachyzoites (2 × 10^6^ per well) were then inoculated into the confluent Vero cell monolayers. At 2 h after infection, the cell monolayers were washed twice with PBS to remove extracellular tachyzoites and then total genomic DNA from the cell samples was isolated with the DNAiso reagent (Takara). The anti-invasion capabilities of NBZ023 and NBZ035 were determined by qPCR. The *T. gondii* RH strain with green fluorescence was seeded as described above. After infection, the cells were incubated for 36 h to fully observe the infection status of the parasites via fluorescence microscopy.

### Ultrastructural analysis

The effects of the incubation with the drugs on the ultrastructure of *T. gondii* tachyzoites were observed via transmission electron microscopy (TEM). For TEM analysis, Vero cells were grown to confluence in T25 flasks and infected with 2 × 10^6^ *T. gondii* tachyzoites for 8 h. After NBZ023 (2.85 μM) or NBZ035 (1.6 μM) was added, the cells were cultured for 8 or 24 h, then digested with TrypLE Express for 2 min, and washed twice with PBS. The detailed experimental procedures were described in a previous study [25–27].

### *In vitro* activity of the compounds against the *T. gondii* PRU strain

Vero cells were infected for 8 h with extracellular type II PRU strain tachyzoites (1.5 × 10^5^), which were isolated from a mouse brain, and then the medium containing compounds or 0.1% DMSO (as a control) was added. After 24, 48, and 72 h of incubation, the numbers of PVs were assessed via electron microscopy under a 40 × objective lens, and at least 20 random fields per well were counted.

The conditions for inducing the formation of cysts *in vitro* by the type II PRU strain were described in a previously published study. After the induction of cyst formation, the alkaline medium was removed and replaced with a medium containing NBZ023, NBZ035, or 0.1% DMSO as the control. After 48 h, the DMEM was removed, and the PRU strain bradyzoite cysts were stained with Dolichos biflorus agglutinin (DBA) to assess the treatment effects.The stained cysts were observed using light microscopy under a 40 × objective lens, with at least 3 random fields per well counted [28].

### *In vivo* efficacy of the compounds against acute* T. gondii* infection in mice

Female BALB/c mice (18‒20 g, 6‒8 weeks old) were divided into 4 groups consisting of 6 mice each, and each mouse was injected intraperitoneally with 2 × 10^3^ Type I tachyzoites. Four hours after inoculation, the treatments were administered once per day for 7 consecutive days. NBZ023 and NBZ035 were dissolved in isotonic saline containing 5% ethanol and 5% Cremophor EL to a concentration of 1 mg/mL for *in vivo* experiments. Sulfadiazine (10 mg/mL, Sigma, USA), pyrimethamine (5 mg/mL, Sigma, USA), and folinic acid (1.5 mg/mL, Sigma, USA) were suspended in isotonic saline containing 1% CMC-Na and used as positive drugs in *in vivo* experiments. The control drugs were administered orally according to the conventional protocol in the references [26], while the novel compounds NBZ023 and NBZ035 were administered intraperitoneally, bypassing their limited gastrointestinal absorption to reach therapeutic plasma concentrations. After the mice were euthanized on the eighth day, the brains, livers, and spleens were collected. DNA was isolated with a DNA purification kit (D3396, Omega Bio-Tek, USA). The *in vivo* efficacy of the compounds against *T. gondii* was evaluated via quantitative real-time PCR with DNA from the brain, liver, and spleen. The numbers of parasites in the tissue samples were determined using standard curves constructed using known numbers of tachyzoites, as previously described [25, 26, 29].

### Statistical analysis

The data were plotted by using the GraphPad Prism 5.0 software (GraphPad Software, Inc., San Diego, CA, USA) and the SPSS 19.0 software (SPSS, Inc., Chicago, IL, USA). The results are presented as the means ± standard deviations (SDs) from at least three independent experiments. The IC_50_ and EC_50_ values were plotted via nonlinear regression analysis (curve fitting) by using the SPSS 19.0 software. The results of the anti-invasion and anti-proliferation tests were compared via one-way analysis of variance in SPSS. The *in vivo* results of the parasite burden and the body weight changes were also analyzed via ANOVA by using GraphPad Prism. Differences in chronic infection burdens were compared via a nonparametric Mann‒Whitney test. Differences were considered statistically significant at a *P* value ≤ 0.05.

## Results and discussion

### Analysis of anti-*T. gondii* activity and cytotoxicity of derivatives

**Fig. 1:**
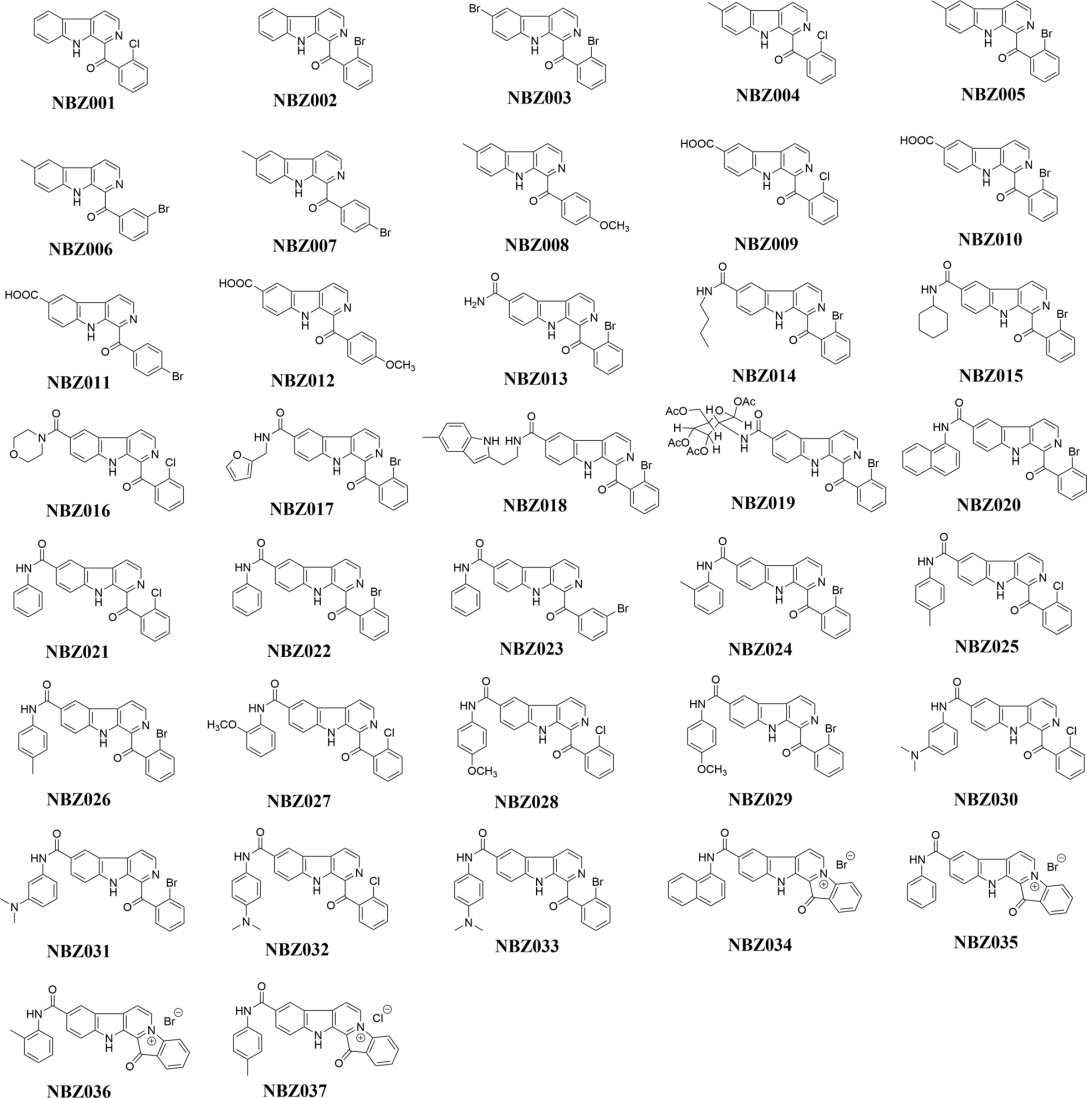
Chemical structures of the β-carboline alkaloid derivatives (NBZ001‒037)

At first, 37 structurally diverse compounds (NBZ001‒037) were screened to examine the influence of their substituents on the activity of these compounds against toxoplasmosis (Fig. 1, Table 1). All β-carboline derivatives generally demonstrated a high level of safety, and the detailed cytotoxicity data of these derivatives are shown in Figure S2. Anti-*T. gondii* activity screening was carried out at 30 μM concentration via plaque and qPCR assays. Pyrimethamine at the EC_50_ was used as the positive control drug. The data revealed that derivatives NBZ011, NBZ018, NBZ021, NBZ023, NBZ027, NBZ028, NBZ030‒032, NBZ034, NBZ035, and NBZ037 had excellent anti-*T. gondii* activities, exceeding 50%, as shown in Table 1. Compared with nonaromatic heterocycles (NBZ016–018), benzannulation enhanced the anti-*T. gondii* activity of NBZ023, showing a 2- to 3-fold increase in potency. Notably, halogen identity at the benzoyl moiety significantly influenced activity: ortho-Cl substitution (NBZ021) improved efficacy by 66.21% over NBZ022. Substituent positioning also proved critical, with meta-Br (NBZ023) exhibiting superior activity to ortho-Br (NBZ022). C6-Phenylformamide substitution improved both the therapeutic index and anti-parasitic efficacy. Subsequent modifications of the phenylformamide moiety with methyl, methoxy, or N, N-dimethylamino groups revealed that N-phenyl methylation and halogen variation (NBZ024–026) induced negligible activity changes. Methoxy-substituted derivatives (NBZ027–029) showed significant potency differentiation, with NBZ028 demonstrating optimal activity (92%). N, N-Dimethylamino-halogen synergism (NBZ030–033) modulated activity moderately to substantially. Finally, fascaplysin-type cyclized β-carbolines exhibited favorable safety profiles and potent anti-*T. gondii* activity (42.88–98.90%, Table 1). Direct comparisons between fascaplysins (NBZ034‒037) and their corresponding parental β-carboline precursors (NBZ020, 022, 024, and 025) were highly important for the analysis of structure–activity relationships. Considering the aspects of safety and anti-*T. gondii* activity, NBZ023 and NBZ035 can be used as lead compounds for subsequent in-depth research, clearly indicating that the features of the molecular scaffolds play key roles in the biological activity against *T. gondii*.

**Table 1:** Anti-*T. gondii* activity and cytotoxicity of β-carboline derivatives NBZ001‒037.

Code	Cytotoxicity (μM)	Inhibition rate of Vero cell at 30 μM (%)	Anti-*T. gondii* activity at 30 μM (%)
NBZ001	> 30.00	24.25	31.27
NBZ002	> 30.00	27.16	42.19
NBZ003	> 30.00	21.84	40.78
NBZ004	> 30.00	5.35	43.79
NBZ005	> 30.00	43.94	33.92
NBZ006	> 30.00	13.06	33.27
NBZ007	> 30.00	13.48	31.21
NBZ008	> 30.00	13.13	17.32
NBZ009	> 30.00	15.01	32.09
NBZ010	> 30.00	16.55	45.24
NBZ011	> 30.00	3.88	61.17
NBZ012	> 30.00	7.66	21.87
NBZ013	> 30.00	11.24	34.50
NBZ014	> 30.00	19.74	2.09
NBZ015	> 30.00	3.68	37.12
NBZ016	> 30.00	10.49	37.39
NBZ017	> 30.00	10.75	43.38
NBZ018	> 30.00	11.76	56.68
NBZ019	> 30.00	9.56	35.90
NBZ020	> 30.00	8.15	7.22
NBZ021	> 30.00	11.45	66.21
NBZ022	> 30.00	46.80	17.38
NBZ023	> 30.00	4.56	99.48
NBZ024	> 30.00	24.38	3.45
NBZ025	> 30.00	3.96	32.58
NBZ026	> 30.00	23.60	21.12
NBZ027	> 30.00	27.90	78.60
NBZ028	> 30.00	19.33	92.00
NBZ029	> 30.00	27.90	30.27
NBZ030	> 30.00	12.78	69.25
NBZ031	> 30.00	6.47	72.65
NBZ032	> 30.00	15.97	73.01
NBZ033	> 30.00	11.18	14.27
NBZ034	> 30.00	58.25	79.00
NBZ035	> 30.00	3.27	98.42
NBZ036	> 30.00	9.02	42.88
NBZ037	> 30.00	29.68	98.90

### The lead compounds NBZ023 and NBZ035 potently inhibit invasion and proliferation of *T. gondii* RH strain

**Fig. 2:**
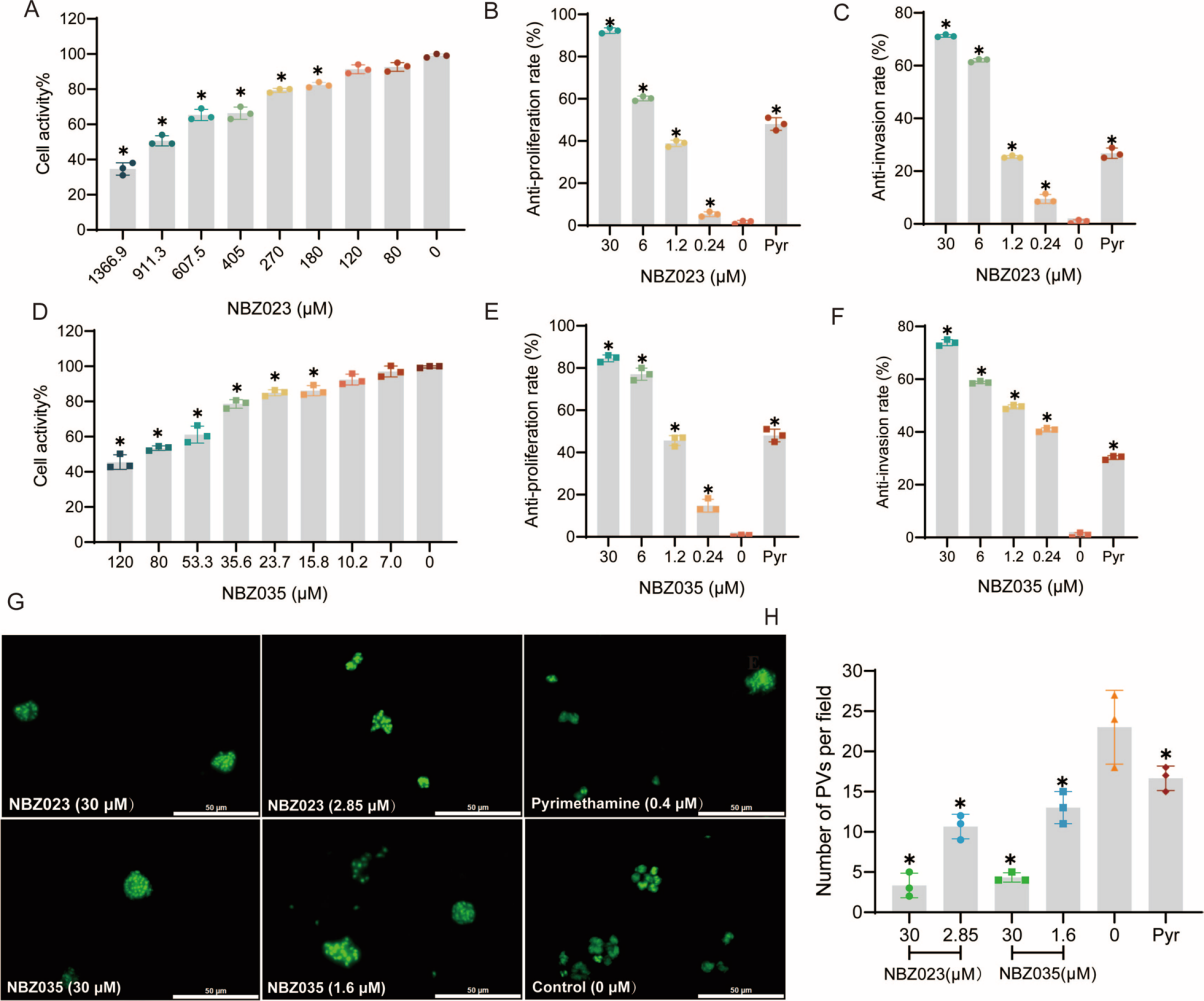
The lead compounds NBZ023 and NBZ035 showed low cytotoxicity and selectively inhibited the growth of RH strain tachyzoites. **A**, **D** The cytotoxicity of NBZ023 and NBZ035 for Vero cells. **B**, **E** NBZ023 and NBZ035 inhibited the proliferation of RH strain tachyzoites in a concentration-dependent manner in the 0‒30 μM concentration range. **C**, **F** NBZ023 and NBZ035 inhibited the invasion of RH strain tachyzoites in a concentration-dependent manner in the 0‒30 μM concentration range. **G** The invasion of the RH-GFP strain treated with NBZ023 and NBZ035 were observed via fluorescence microscope in 10 random fields per well. Magnification, 40 × ; scale bars, 50 μm. The red arrow points to a PV formed by the RH-GFP strain. All the data are presented as the mean ± SDs from three replicate experiments. *P ≤ 0.05 compared with the control. **H** The number of PVs in each random field was significantly reduced, indicating that NBZ023 and NBZ035 significantly inhibited *T. gondii* invasion

To evaluate the selectivity index (SI) of the lead compound against *T. gondii*, the cytotoxicity of NBZ023 and NBZ035 to host cells was examined via the CCK-8 assay, and the half-maximal cytotoxic concentration (CC_50_) against host cells was found to be 883 μM and 80.58 μM, respectively (Fig. 2A, D). To further quantify their efficacy, qPCR analysis was conducted. The qPCR results revealed that NBZ023 and NBZ035 inhibited *T. gondii* proliferation in a concentration-dependent manner, with the IC_50_ of 2.85 μM and 1.6 μM, and the SI values of 309.8 and 50.86, respectively (Fig. 2B, E). Compared with recent findings for other β-carboline alkaloids (norharmane, harmane and harmine) that exhibit activity against *T. gondii,* our findings suggested that the activity and safety range of NBZ023 and NBZ035 were significantly better. The SI of NBZ023 and NBZ035 were 18-fold and threefold greater than that of 9-methyl-harmine (SI = 17.2), respectively [16]. Furthermore, NBZ023 and NBZ035 obviously suppressed the invasion of *T. gondii* in a concentration-dependent manner (*P* ≤ 0.05), with the IC_50_ values of 4.72 μM and 1.13 μM, respectively. The anti-invasion rate of pyrimethamine (0.4 μM) at the IC_50_ was 26.77% (Fig. 2C, F). Additional results were obtained via fluorescence microscopy, as shown in Fig. 2G and H. Compared with those in the control and positive control groups, the number of parasitophorous vacuoles (PVs) invaded by tachyzoites of the RH-GFP strain was significantly reduced after NBZ023 or NBZ035 treatment. Thus, derivative NBZ023 and NBZ035 can significantly inhibit the proliferation and invasion of the *T. gondii* RH strain.

### The lead compounds NBZ023 and NBZ035 induce ultrastructural alterations in   *T. gondii*

**Fig. 3:**
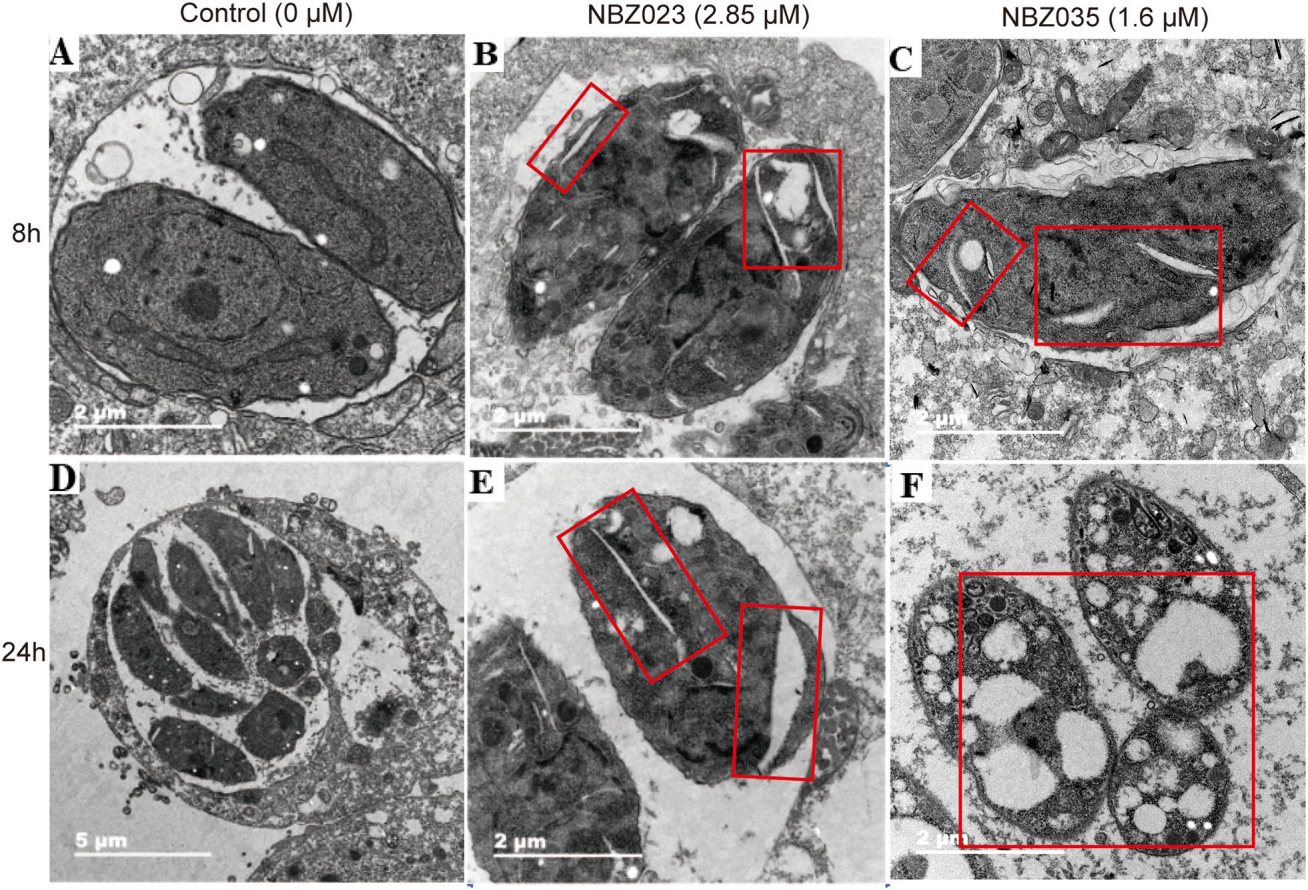
Ultrastructural changes in *T. gondii* tachyzoites after NBZ023 and NBZ035 treatment. **A**, **D** In the control group, *T. gondii* proliferated in the form of endodyogeny within PVs and had a complete plasma membrane and the nucleus. **B**, **C** After treatment with NBZ023 and NBZ035 for 8 h, obvious cavitation and cracks appeared in the *T. gondii* tachyzoites. **E**, **F** After 24 h, NBZ023- and NBZ035-induced tachyzoite vacuolation and cracks were more obvious. The red boxes indicate *T. gondii* vacuolation and cracks. Scale bars: 2 μm (**A**, **C**–**F**) and 5 μm (**B**)

The lead compounds NBZ023 and NBZ035 change the ultrastructure and cause apoptosis of *T. gondii*. The results revealed that *T. gondii* tachyzoites in the control group proliferated into a banana-like morphology and formed PVs surrounded by host cell mitochondria after 8 or 24 h (Fig. 3A, D). NBZ023 and NBZ035 both caused cracks in the somatic cytoplasm of the parasite after incubation for 8 h (Fig. 3B, C). After 24 h of treatment, the parasite ruptured, and severe cavitation occurred (Fig. 3E, F). These drastic cellular disruptions could indicate cell death by apoptosis, as it has been previously reported that the cleft is one of the markers of apoptotic cell death [30].

### The lead compounds NBZ023 and NBZ035 inhibit cyst formation by the PRU strain

**Fig. 4:**
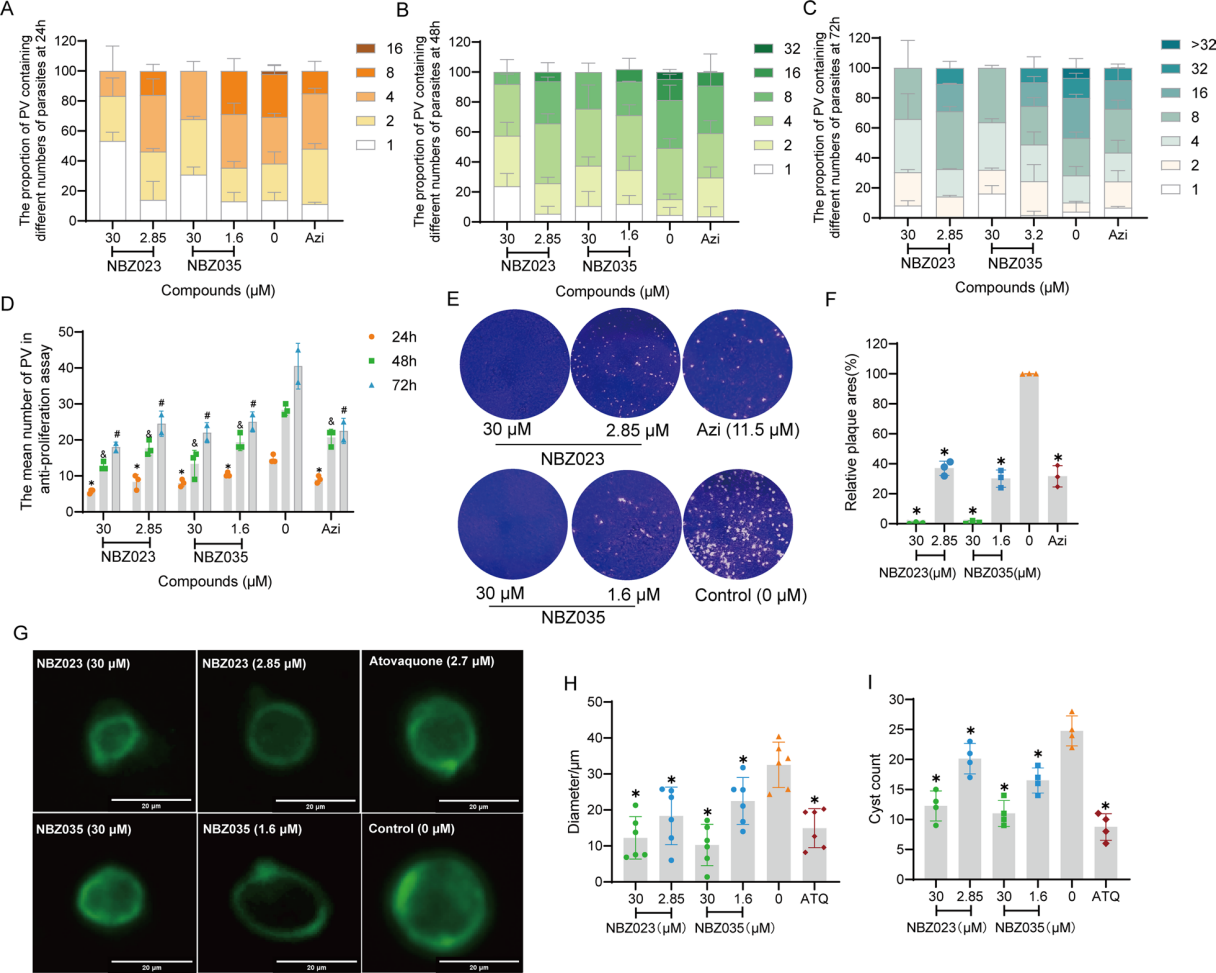
The lead compounds NBZ023 and NBZ035 significantly inhibited the proliferation of PRU tachyzoites and the formation of cysts. **A**‒**C** After treatment with the lead compounds NBZ023 (2.85 or 30 μM) or NBZ035 (1.6 or 30 μM) for 24 h, 48 h and 72 h, the number of tachyzoites of the PRU strain in each PV was counted under a 40 × objective lens in at least 20 random fields per well. **D** The number of PVs of the *T. gondii* PRU strain was counted in each random field after 24, 48, and 72 h of incubation, with at least 20 random fields counted per well. The data suggested that the number of PVs decreased significantly after 24, 48, and 72 h of incubation. *P ≤ 0.05 compared with the control group at 24 h; &*P* ≤ 0.05 compared with the control group at 48 h; ^#^*P* ≤ 0.05 compared with the control group at 72 h. **E**‒**F** The numbers and areas of plaque were used to evaluate the activity of NBZ023 and NBZ035 against PRU strain tachyzoites. The data were compiled from three independent experiments, and the error bars represent standard errors of the means (SEMs). *P ≤ 0.05. **G** Dolichos biflorus agglutinin staining of the cyst cell wall. A fluorescence microscope was used to observe at least 3 random fields per well. Magnification, 40 × ; scale bars, 20 μm. **H** The cysts in each random field were significantly smaller after NBZ023 and NBZ035 treatment. **I** The number of cysts in each random field was also significantly reduced after NBZ023 and NBZ035 treatment. Three independent experiments were performed, and the data are expressed as the means ± SDs, *P ≤ 0.05

We further evaluated the anti-*T. gondii* effect of lead compounds on the PRU strain. To our delight, NBZ023 and NBZ035 significantly (*P* ≤ 0.05) inhibited the proliferation ability of the *T. gondii* PRU strain, and the number of parasites per PV and the number of PVs decreased significantly (*P* ≤ 0.05) in a time-dependent manner after 24, 48, and 72 h of incubation (Fig. 4A‒D). Compared with those in the control group, the number and area of plaques formed by PRU strain-infected Vero cells were also significantly reduced (Fig. 4E, F).

Furthermore, the anti-*T. gondii* effect of NBZ023 and NBZ035 on the PRU cysts was evaluated in Vero cells *in vitro*, as illustrated in Fig. 4G‒I. Compared with the control, NBZ023 (2.85 or 30 μM) and NBZ035 (1.6 or 30 μM) significantly (*P* ≤ 0.05) inhibited cyst formation, and the size and number of cysts were markedly (*P* ≤ 0.05) reduced. By contrast, NBZ023 and NBZ035 significantly inhibited the formation of *T. gondii* cysts by twofold compared with that in the control group, and the inhibitory effect was equivalent to that of the positive control drug atovaquone. Previous studies reported that the histone deacetylase inhibitor FR235222 could reduce the interconversion of bradyzoites and tachyzoites and the number of cysts *in vitro*, but its inhibitory activity was limited [31]. These findings suggest that NBZ023 and NBZ035 are potential candidate compound for treating chronic *T. gondii* infection, but its mechanism of action needs to be further explored.

### The lead compounds NBZ023 and NBZ035 have potencies in preventing toxoplasmosis in a murine model

**Fig. 5:**
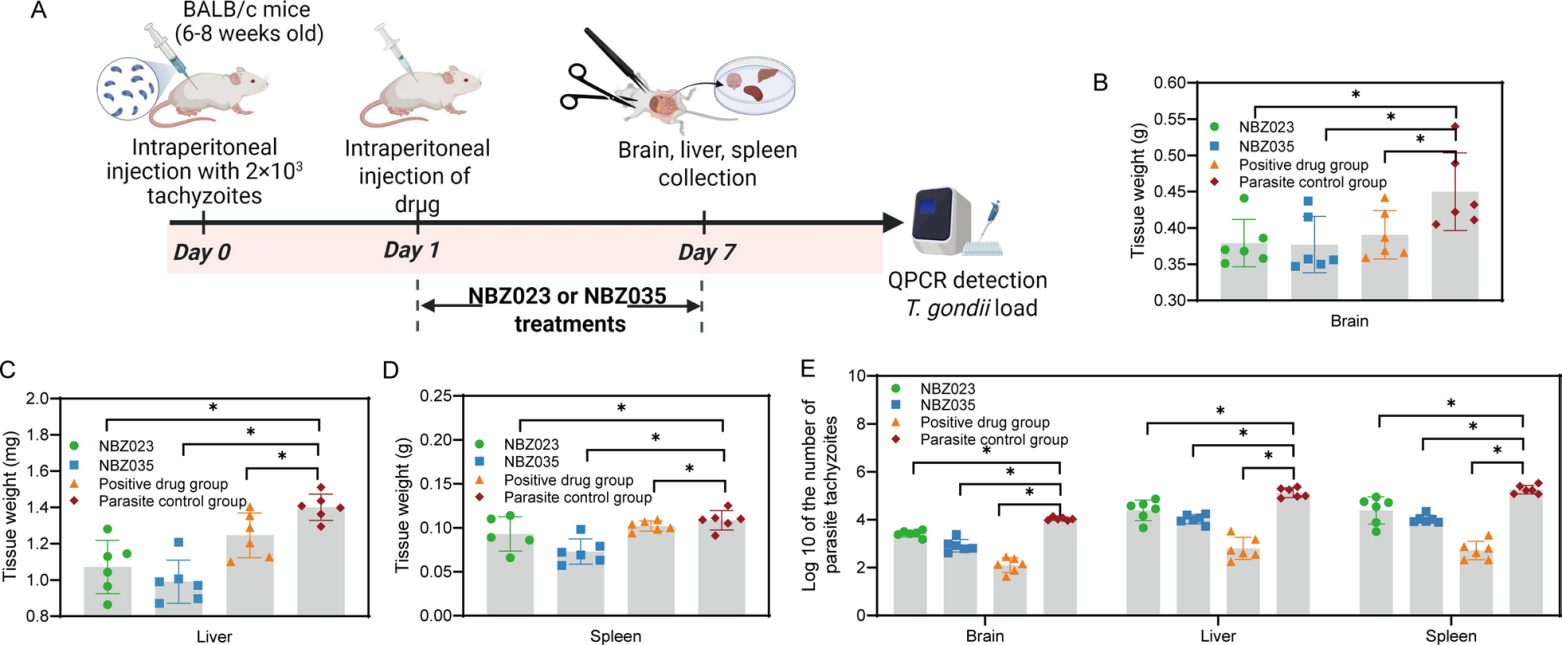
The lead compounds NBZ023 and NBZ035 treat the acute RH strain infection mice *in vivo* (**A**). **B**‒**D** Brain, liver, and spleen tissue weights. **E** NBZ023 and NBZ035 treatments reduced the parasite burden in tissues from acutely infected mice. The mice were challenged intraperitoneally with *T. gondii* tachyzoites and then treated with NBZ023, NBZ035 or PBS once daily for 7 days. The parasites from the brain, liver, and spleen tissues of the infected BALB/c mice were isolated and homogenized. Total genomic DNA was isolated, and the level of the *T. gondii* 529-bp gene was quantified via qPCR. The quantified parasite loads in the tissues of the mice are presented as the log10 values of the number of tachyzoites per 25 mg of tissue. *P ≤ 0.05 compared with the parasite control

NBZ023 and NBZ035 were tested in a murine model of acute infection, as shown in Fig. 5A. Compared with those in the parasite control groups, the brain, liver, and spleen were significantly  smaller in the treated group (Fig. 5B‒D). Compared with the parasite control group, treatment with NBZ023, NBZ035 or the positive control significantly (*P* ≤ 0.05) reduced the quantities of *T. gondii* in the brain, liver and spleen tissues, as shown in Fig. 5E. However, NBZ035 was much more efficacious than NBZ023 at reducing the *T. gondii* burden in the brain, liver, and spleen. Compared with that in the parasite control group, the mean *T. gondii* burden in the liver was reduced by NBZ023 and NBZ035, which were administered at 10 mg/kg BW, by 56.42% and 88.52%, respectively. Similarly, NBZ023 and NBZ035 reduced the mean numbers of *T. gondii* parasites by 70.72% and 93.21%, respectively, in the brain and by 68.90% and 89.40%, respectively, in the spleen, which suggests that during active infection, NBZ035 achieves brain concentrations that are sufficient to inhibit *T. gondii* replication or that NBZ035 prevents brain infection progression by inhibiting systemic toxoplasmosis. The positive control group had a greater than 98% reduction in the mean burden of *T. gondii* in the brain, liver, and spleen. These experiments demonstrated that NBZ035 was highly active against acute toxoplasmosis in mice.

## Conclusions

In summary, a series of β-carboline derivatives were synthesized and systematically evaluated to explore their therapeutic potential against toxoplasmosis. Lead compounds NBZ023 and NBZ035 exhibited an excellent overall efficacy against the *T. gondii* RH and PRU strains, and were highly effective at preventing toxoplasmosis during murine infection, which are expected to be developed as new anti-toxoplasmosis drugs.

## Supplementary Information

Supplementary material 1.

## Data Availability

All data and materials generated or analyzed in this study have been provided in the article.

## Abbreviations

PRU: Prugniaud
Pyr: Pyrimethamine
Sul: Sulfadiazine
βCs: β-Carboline alkaloids
NHPI: N-hydroxyphthalimide
HBTU: *O*-(Benzotriazol-1-yl)-*N*, *N*, *N’*, *N*’- tetramethyluronium hexafluorophosphate
SAR: Structure–activity relationship
IC_50_: The half inhibition concentration
SI: Selectivity index
PVs: Parasitophorous vacuoles
HPLC: High performance liquid chromatography
HRMS: High-resolution mass spectrometry
ESI: Electrospray ionization
FBS: Fetal bovine serum
Vero: African green monkey kidney
NEAA: Non-essential amino acids
RH-GFP: *T. gondii* RH strain parasites, constitutively expressing nuclear green fluorescent protein
TEM: Transmission electron microscopy
ANOVA: Analysis of variance
