# Supplementary material for: Design, synthesis, and anti-Toxoplasma gondii evaluation of β-carboline derivatives
Source: Parasit Vectors. 2025 Dec 29;18:507. doi: 10.1186/s13071-025-07139-6 (PMC12750888; doi:10.1186/s13071-025-07139-6)
Supplement: Supplementary file 1 — Supplementary material 1. [file 13071_2025_7139_MOESM1_ESM.docx]

**Supplementary Information**

**Design, Synthesis, and Anti-Toxoplasma gondii Evaluation of β-Carboline Derivatives**

Zhendi Liu^a,e^, Yongmei Li^b^, Yetian Li^a^, Xiaoyu Han^a^, Hongda Qiu^b^, Chang Qin^a^, Yuchao Zhu^d^, Weida Liang^b^, Jiao Mo^a^, Zixun Yan^a^, Weixin Gao^a^, Jiyu Zhang^c^, Jishan Zheng^e^, Hongze Liang^b*^, Jili Zhang^a*^

^a^ School of Basic Medical Sciences, Health Science Centre, Ningbo University, 315211, Ningbo, China；

^b^ Key Laboratory of Advanced Mass Spectrometry and Molecular Analysis of Zhejiang Province, School of Materials Science and Chemical Engineering, Ningbo University, 315211, Ningbo, China；

^c^ Lanzhou Institute of Husbandry and Pharmaceutical Sciences, Chinese Academy of Agricultural Sciences, 730046, Lanzhou, China;

^d^ Department of Radiology First Affiliated Hospital of Ningbo University, 315010, Ningbo, China.

^e^ Ningbo Women and Children's Hospital, Ningbo, Zhejiang 315012, China.

* Corresponding author:

Ningbo University, 315211, Ningbo, People’s Republic of China, E-mail address: [zhangjili@nbu.edu.cn](mailto:zhangjili@nbu.edu.cn)；

Key Laboratory of Advanced Mass Spectrometry and Molecular Analysis of Zhejiang Province, School of Materials Science and Chemical Engineering, Ningbo University, 315211, Ningbo, People’s Republic of China, E-mail address: [lianghongze@nbu.edu.cn](mailto:lianghongze@nbu.edu.cn).

**List of contents**

**[Figure S1.](#_Toc7867)** [The synthesis schemes and mass spectra of NBZ027 and NBZ037.............................. 3](#_Toc7867)

**Figure S2.** The detailed cytotoxicity data of NBZ001-NBZ037......................................................5

**[Scheme S1.](#_Toc27888)** [Synthesis of β-carboline derivatives NBZ001–026 and NBZ028-NBZ033 6](#_Toc27888)

**[Scheme S2.](#_Toc13548)** [Synthesis of β-carboline derivatives NBZ034–36 6](#_Toc13548)

**[Scheme S3.](#_Toc13548)** [Synthesis of β-carboline derivatives NBZ027 and NBZ037 6](#_Toc13548)

**
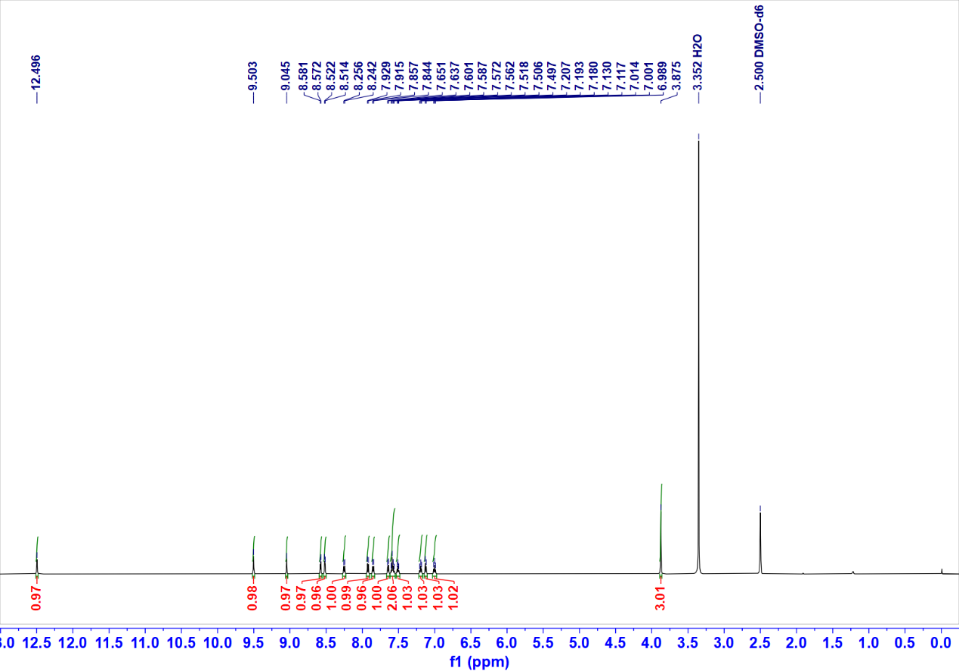
**

**^1^H NMR of NBZ027**

**
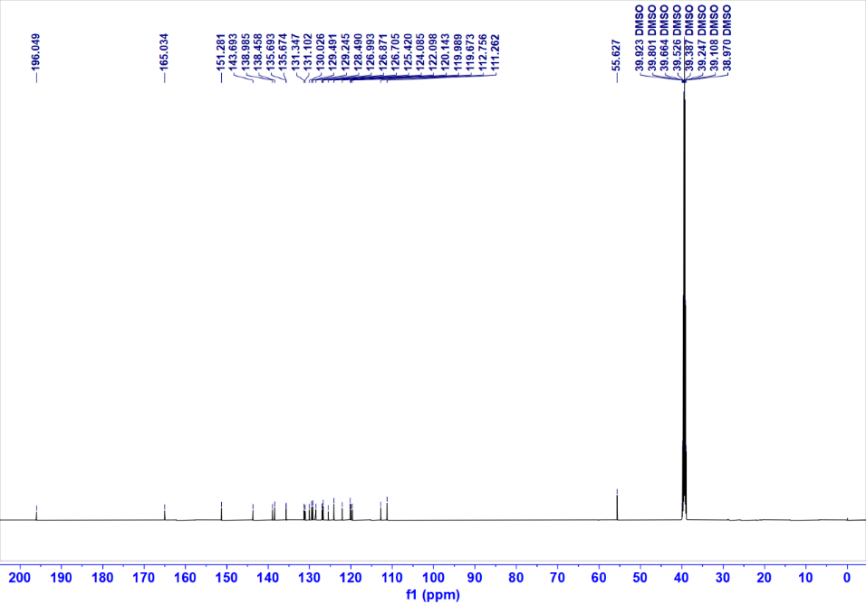
**

**^13^C NMR of NBZ027**

**HRMS of NBZ027**


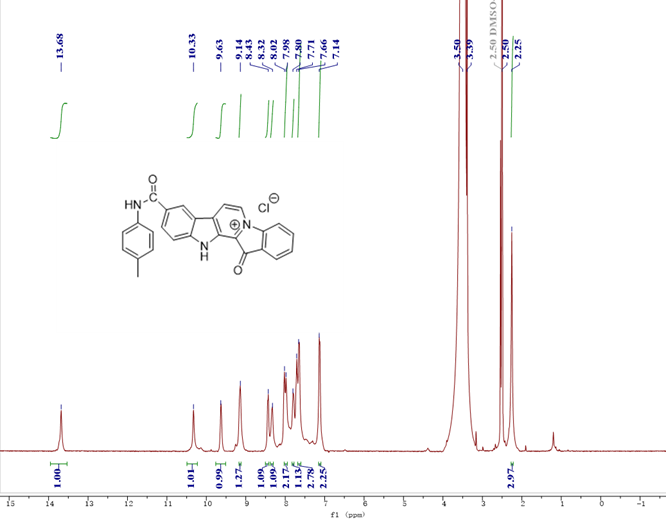


**^1^H NMR of NBZ037**


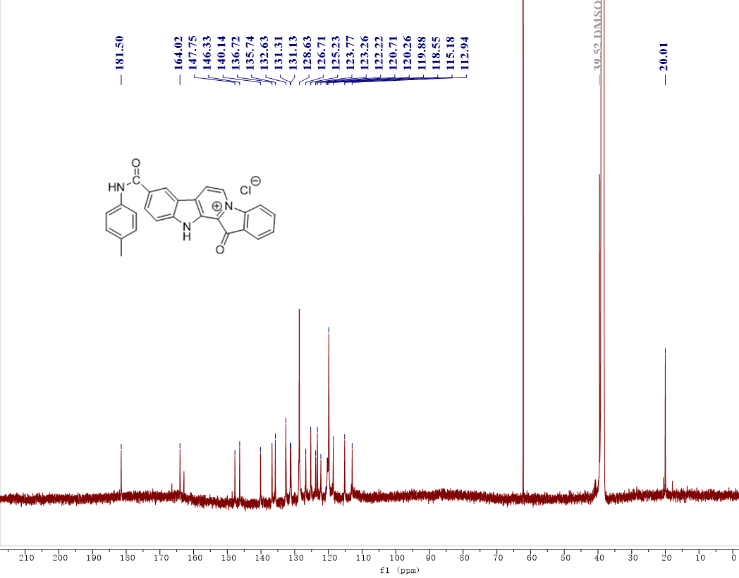


**^13^C NMR of NBZ037**

**HRMS of NBZ037**

# Figure S1. The synthesis schemes and mass spectra of NBZ027 and NBZ037.

#
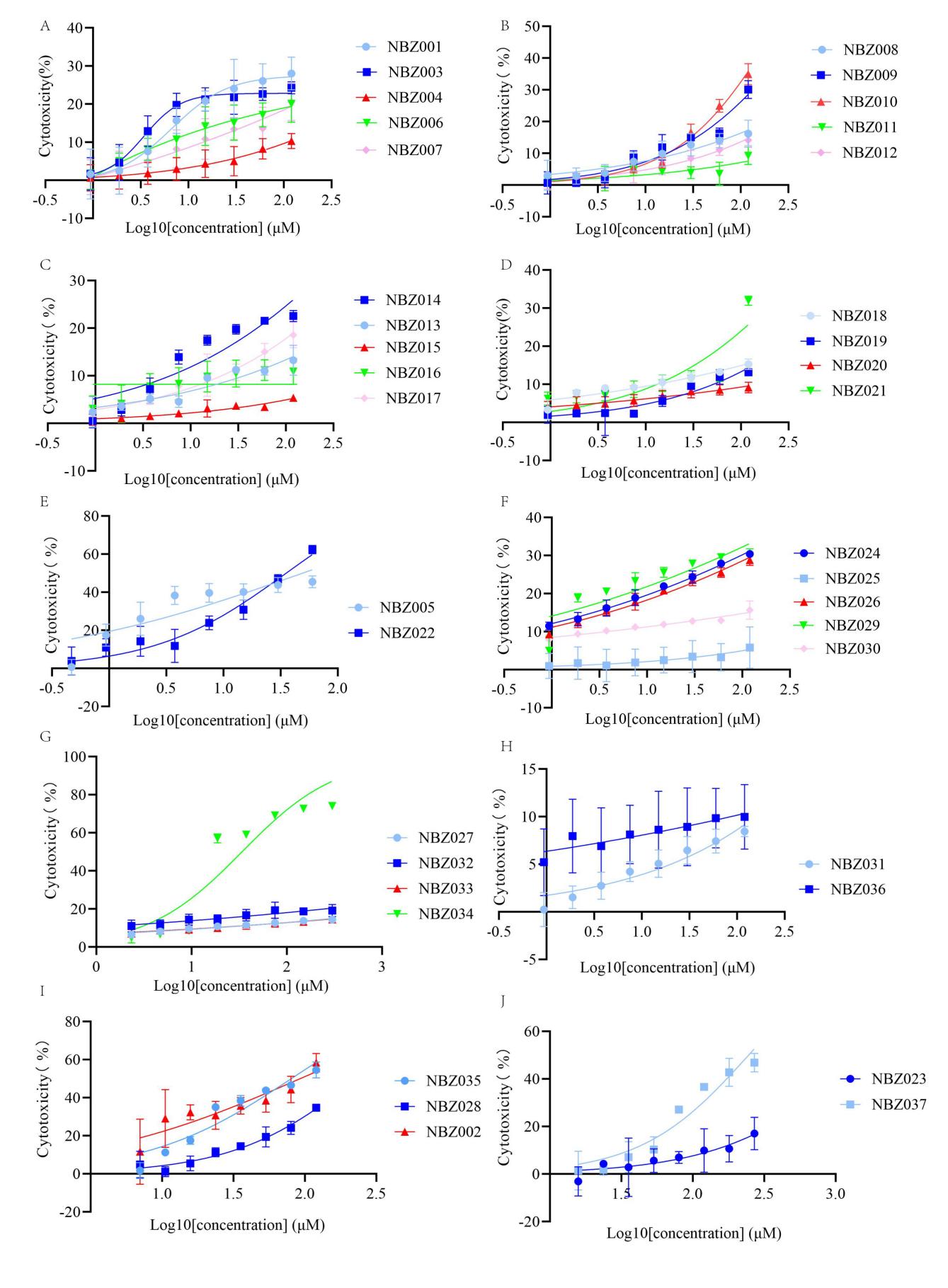
Figure S2. The detailed cytotoxicity data of NBZ001-NBZ037.


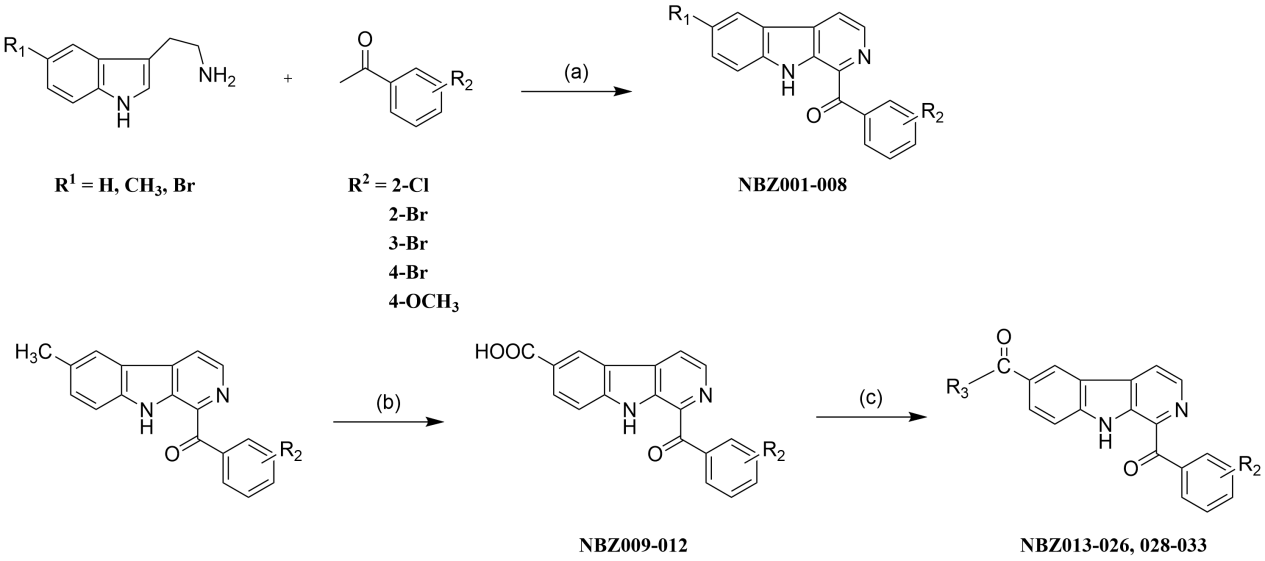


Scheme S1. Synthesis of β-carboline derivatives NBZ001–026 and NBZ028-NBZ033**.** Reagents and conditions: (a) I_2_, DMSO, reflux, 2 h; (b) O_2_, NHPI, Co (OAc)_2_, CH_2_Cl_2_, room temperature, 6 h; (c) HBTU, Et_3_N, amines, DMF, 30–75 ℃, 5 h for NBZ0013–026 and NBZ028–033.


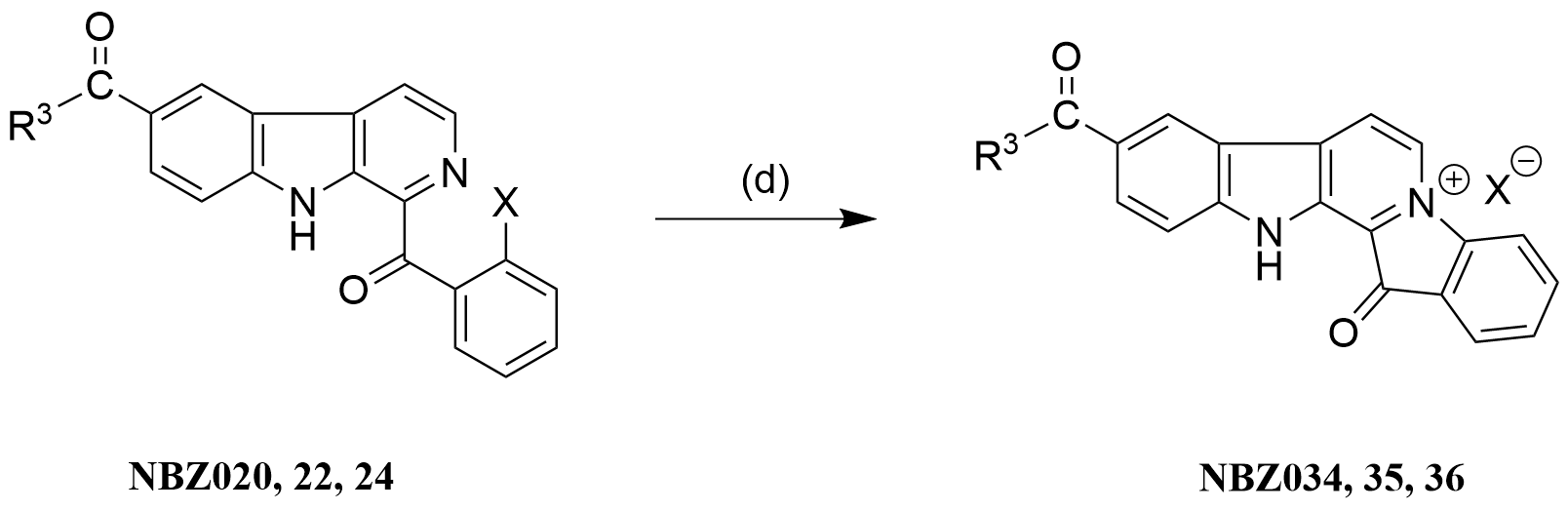


Scheme S2. Synthesis of β-carboline derivatives NBZ034–36. Reagents and conditions: (d) N_2_, ethylene glycol or decahydronaphthalene, 210–220 ℃, 1.5 h.


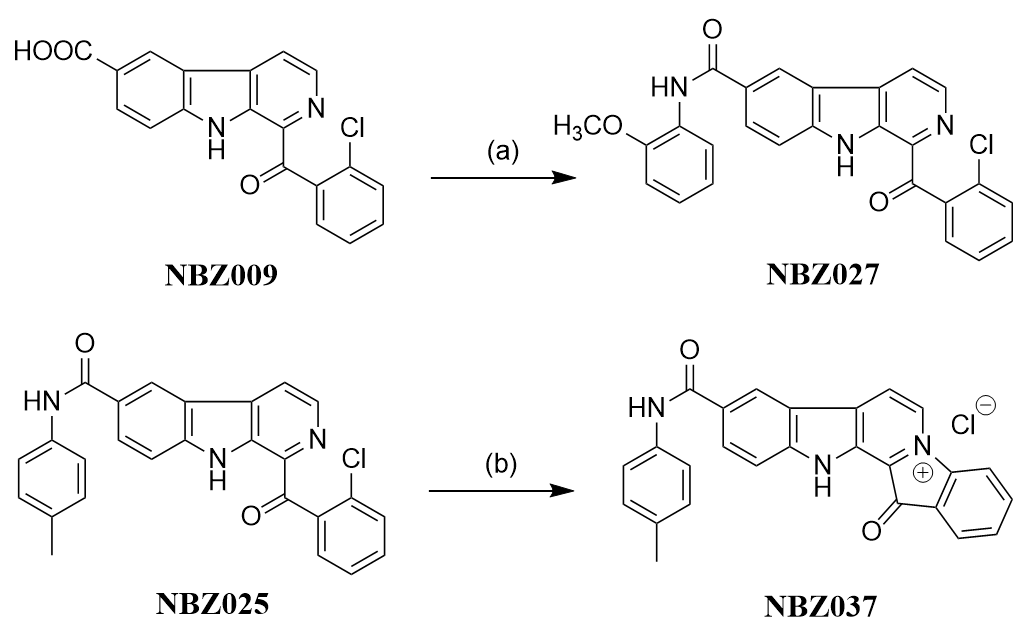


**Scheme S3**. Synthesis of β-carboline derivatives NBZ027 and NBZ037. Reagents and conditions: (a) HBTU, Et_3_N, amine, DMF, 30 ℃, 5 h for NBZ027; (b) N_2_, ethylene glycol or decahydronaphthalene, 210–220 ℃, 1.5 h for NBZ037.
